# Calcined low-grade phosphate rock fertilization enhances nitrogen fixation, yield, and grain quality in soybeans

**DOI:** 10.3389/fpls.2025.1581961

**Published:** 2025-06-19

**Authors:** Andressa Nakagawa, Papa Saliou Sarr

**Affiliations:** Crop, Livestock and Environment Division, Japan International Research Center for Agricultural Sciences, Tsukuba, Japan

**Keywords:** soybean, phosphate rock, root nitrogen fixation, seed protein, seed lipid

## Abstract

The limited use of chemical fertilizers in developing countries has posed a significant challenge to sustainable crop production. Beyond increasing yields, improving seed nutritional quality is also crucial. This study evaluated the impact of phosphorus (P) fertilization, using calcined low-grade phosphate rock, on soybean growth, yield, and seed quality. Soybean cultivars, Fukuyutaka and Jenguma were grown under three treatments: no P application (–P), triple superphosphate [+P(TSP)], and calcined phosphate rock [+P(PR)]. Both P treatments significantly increased pod number (e.g., 12 pods plant−¹ in (–P) vs. 25 pods plant−¹ in +P(TSP) for Fukuyutaka), seeds number (23 vs. 48 seeds plant−¹), and seed yield (5.03 g vs. 14.51 g plant−¹) compared to the control. However, P fertilization only increased the average individual seed weight in Fukuyutaka. P application also enhanced root nodulation–nodule numbers in Jenguma increased from 22 in (–P) to 102 in +P(PR)–and boosted nitrogen (N) fixation in both cultivars. Shoot dry weight doubled under P fertilization, accompanied significant increases in shoot N and P contents. Seed composition responses varied by cultivar: in Fukuyutaka, P application reduced protein content but increased lipid content, while in Jenguma, P fertilization increased protein content and had little to no effect on lipid content. Overall, the results demonstrate that calcined phosphate rock is an effective and affordable alternative to triple superphosphate for improving soybean growth, nodulation, nitrogen fixation, and seed quality. It offers a promising phosphorus source for smallholder farmers in phosphorus-deficient soils of sub-Saharan Africa.

## 1 Introduction

Soybean (*Glycine max* L. Merr.) is a globally important legume, valued for its high protein content and edible oil, making it a key crop for the food and feed industries (Song et al., 2023). In 2023, global soybean production reached 394.714 million metric tons (USDA, 2024). However, with a growing global population, increasing soybean yield remains a critical challenge, especially in sub-Saharan Africa (SSA). SSA faces significant constraints, including land degradation and low soil fertility, to meet its increasing food demands. The region’s population is projected to increase from 1.3 billion in 2019 to 2.5 billion by 2050 (United Nations, 2019), highlighting the urgent need for sustainable agricultural practices.

Biological Nitrogen fixation (BNF) plays a crucial role in legume productivity by forming a symbiotic relationship between nitrogen-fixing bacteria and plant roots, thereby enhancing plant growth and increasing seed yield (Wang et al., 2018). BNF also improves soil health by reducing the need for nitrogen fertilizers (Ladha et al., 2022; Herridge and Rose, 2000; Freitas et al., 2022). However, beyond nitrogen, other soil nutrients, particularly phosphorus (P), are essential to support BNF and overall legume productivity and seed protein content (Ciampitti et al., 2021). P is vital for nodule formation and function (Bargaz et al., 2018; Rotaru and Sinclair, 2009) and is the second most limiting macronutrient in soybean cultivation after nitrogen (Faozi et al., 2019). P deficiency can severely limit soybean growth by reducing plant biomass, nitrogen content, and nitrogen metabolism activity (Davito and Sadras, 2014; Staniak et al., 2024; Sa and Israel, 1995). The high P demand of nitrogenase activity in BNF further exacerbates this challenge (Rycher and Randall, 2006; Wanke et al., 1988; Qiu and Israel, 1994). Soybean nodules under P deficiency have been reported to exhibit reduced carbohydrate (sucrose, hexose) levels and energy status (Li et al., 2022a; Sa and Israel, 1991; Rychter et al., 1992).

In SSA, low soil P availability is a major constraint on crop production (Verde and Matusso, 2014; Stewart et al., 2020). Although phosphate rock (PR) is abundant in many African countries, its low solubility often limits its effectiveness as a direct fertilizer (Sarr et al., 2020). Recent research has focused on calcination of low-grade PR with sodium carbonate (Na_2_CO_3_) to enhance P availability. For instance, calcined PR from Burkina Faso, prepared by blending PR with 30% Na_2_O sourced by Na_2_CO_3_ at 950°C, showed improved solubility, with citric acid soluble P increasing from 31.1% to 97.5% and water-soluble P increasing from 0.2% to 28.1% (Nakamura et al., 2015). The calcined PR contains 19.72% P_2_O_5_ as reported by Nakamura et al. (2015). This suggests that calcined PR could be a viable, cost-effective alternative to chemical fertilizers like triple superphosphate (TSP). Therefore, this study aimed to evaluate the effectiveness of calcined PR compared with TSP in improving soybean growth, nodulation, nitrogen fixation, and seed quality in pot culture experiments using two soybean cultivars.

## 2 Materials and methods

### 2.1 Plant materials, fertilization, and sampling

Soybean seeds of the Japanese cultivar ‘Fukuyutaka’ and the Ghana cultivar ‘Jenguma’, provided by the Council for Scientific and Industrial Research (CSIR)-Savanna Agriculture Research Institute (SARI) in Ghana, were sown in 1/5000 Wagner pots filled with soil collected from the Japan International Research Center for Agricultural Sciences (JIRCAS) experimental field station in Tsukuba, Japan (36°05’N, 140°08’E). The soil, classified as a humic haplic Andosol, had not been fertilized with P for more than 15 years. It has a pH of 5.8 and with a high P-fixing capacity, resulting in a low level of plant-available P (5 mg P kg^-1^ soil, Bray-II), as reported by Ranaivo et al. (2022). The experiment was laid in a randomized complete bloc design with two cultivars, three fertilization treatments, and four replications each. Soil fertilization was performed according to Nakagawa et al. (2020), with differential application of N, P, and K. The ‘-P’ treatment consisted of N (8.8 kg ha^-1^) and K application (10 KCl kg ha^-1^), without P. In the ‘+P (TSP)’ treatment, the same rates of N and K were applied, and P was supplied as triple superphosphate (TSP), providing 22.9 kg P_2_O_5_ ha^-1^. This corresponded to an application of 54.5 kg TSP ha^-1^ (42% P_2_O_5_). The ‘+P (PR)’ treatment, hereafter referred to as PR, also received the same amounts of N, P, and K; however, P was supplied using PR, applied at 127 g PR ha^-1^. Seeds were sown the following day after fertilizer application, and the pots that contained one plant each, were placed into an isolation greenhouse (26/23°C day/night) at JIRCAS and regularly watered until sampling by an automatic watering machine (SAFETY3 SAW-2, Fujihara Industrial Co., Ltd.) for 1 minute twice a day, which was subsequently increased to four times a day from the R1 flowering stage. Shoot, root, and root nodules per replicate were sampled at the seed filling stage, two weeks after the plants reached the R5.5 phenological stage as described by Fehr et al. (1971), and seeds were sampled at harvest during the R8 phenological stage. The plant phenotypes at the time of sampling are shown in **Supplementary Figure S1**.

### 2.2 Soybean agronomic traits, and contents of nitrogen and phosphorus

The plant samples collected were oven-dried at 80°C for two days and ground using a high-speed vibration mill. The shoot and root dry weights were recorded, and the number of nodules was counted at the time of sampling. Seed number and weight were also recorded at harvest. The total carbon and nitrogen contents in both the shoots and roots were determined via the dry combustion method with an NC analyzer (Sumigraph NC 220F, Sumika, Japan). For P content, ground plant samples were dry ashed at 550°C in a muffle furnace and then extracted with 0.5 M hydrochloric acid. The P concentration in the extract was measured by the inductively coupled plasma atomic emission spectrometry (ICPE-9820, Shimadzu, Japan).

### 2.3 Nitrogen fixation quantification and estimation of seed lipid and protein content in soybean

During plant growth (at the R5.5 phenological stage), xylem sap samples were collected from 8:00 to 10:00 a.m. by cutting the stem just above the first node and inserting a silicon tube into the xylem to extract the sap frozen at -20°C until analysis. Nitrogen derived from the atmosphere (Ndfa), representing nitrogen fixation, was quantified using the ureide (N solute) method (Unkovich et al., 2008). Briefly, the method involves boiling the samples in a water bath twice. In the first boiling, 0.5 N NaOH was added to the samples, and 0.65 N HCl/phenyl hydrazine was added in the second boiling. After the samples were cooled in an ice bath, HCl/KFeCN was added. The abundance of the samples was then measured at 525 nm using a Bio-Rad SmartSpec 3000 UV/Vis spectrophotometer (California, USA). At harvest, the seeds were oven-dried at 80°C for two days and then ground in a mill for lipid and protein content analysis. Lipid was extracted using hexane and analyzed following the method described by Saldivar et al. (2011). The nitrogen content was determined by the Kjeldahl method (Jackson, 1973), and the protein content was calculated by multiplying the nitrogen content by 6.25, with the average nitrogen content of proteins being approximately 16% (1/0.16 = 6.25) (Mariotti et al., 2008). All the seeds were harvested at maturity and oven-dried to measure the dry weight, pod number per plant, and total seed yield.

### 2.4 Statistical analysis

Two-way analysis of variance (ANOVA) was conducted to assess the main and interactive effects of P fertilization and soybean cultivar on the measured variables. When significant interactions were observed, one-way ANOVA was performed by cultivar, followed by *post-hoc* multiple comparisons of means by the Tukey’s honestly significant difference (HSD) test, with the significance set at *p* < 0.05. These analyses were conducted using XLSTAT^®^ software (version 2022.4.1.1368; Addinsoft, New York).

## 3 Results

### 3.1 Effect of phosphorus application on soybean growth

Two-way ANOVA revealed a significant interaction effect between P fertilization and soybean cultivar, justifying the presentation of the agronomic data by cultivar (**Table 1**). Pod number, seed number, seed yield, and seed weight of both cultivars were significantly influenced by phosphorus fertilization. The +P (TSP) and +P (PR) treatments showed significantly higher values for all measured parameters compared to the –P treatment (*p* < 0.05). No significant differences were observed between the +P (TSP) and +P (PR) treatments for any parameter. However, the +P (PR) treatment increased seed yields threefold in Fukuyutaka (**Table 1A**) but only twofold in Jenguma (**Table 1B**). Fukuyutaka produced slightly greater seed weight than Jenguma in all P treatments. In contrast, Jenguma produced more pods and seeds than Fukuyutaka, regardless of the P application level.

**Table 1:** Yield components of the cultivars Fukuyutaka (A) and Jenguma (B) under different phosphorus treatments.

(A) cv. Fukuyutaka				
Treatments	Pod number (plant ^-1^)	Seed number (plant ^-1^)	Seed yield (g plant ^-1^)	Seed weight (g seed ^-1^)
- P	12.25 ± 1.32 b	23 ± 2.34 b	5.03 ± 0.46 b	0.22 ± 0.01 b
+ P (TSP)	25.25 ± 2.53 a	48 ± 4.02 a	14.51 ± 0.98 a	0.31 ± 0.01 a
+ P (PR)	22.25 ± 4.42 a	42 ± 8.45 a	13.15 ± 2.44 a	0.31 ± 0.01 a

(B) cv. Jenguma				
Treatments	Pod number (plant ^-1^)	Seed number (plant ^-1^)	Seed yield (g plant ^-1^)	Seed weight (g seed ^-1^)
- P	21.2 ± 2.65 b	36.2 ± 5.75 b	3.43 ± 0.66 b	0.10 ± 0.01
+ P (TSP)	41.5 ± 8.41 a	68.8 ± 16.7 a	8.87 ± 2.35 a	0.11 ± 0.02
+ P (PR)	41.0 ± 2.93 a	73.6 ± 7.87 a	7.13 ± 1.06 a	0.10 ± 0.01

Data are means ± standard error (n = 4). For each cultivar, means within each column followed by different letters are significantly different at *p* < 0.05 (ANOVA followed by Tukey’s HSD test). If no letter differences are present, differences are not statistically significant. P, phosphorus; TSP, triple superphosphate; PR, calcined phosphate rock; Cv, cultivar.

### 3.2 Shoot and root dry weights and their nitrogen and phosphorus contents

**Figure 1:**
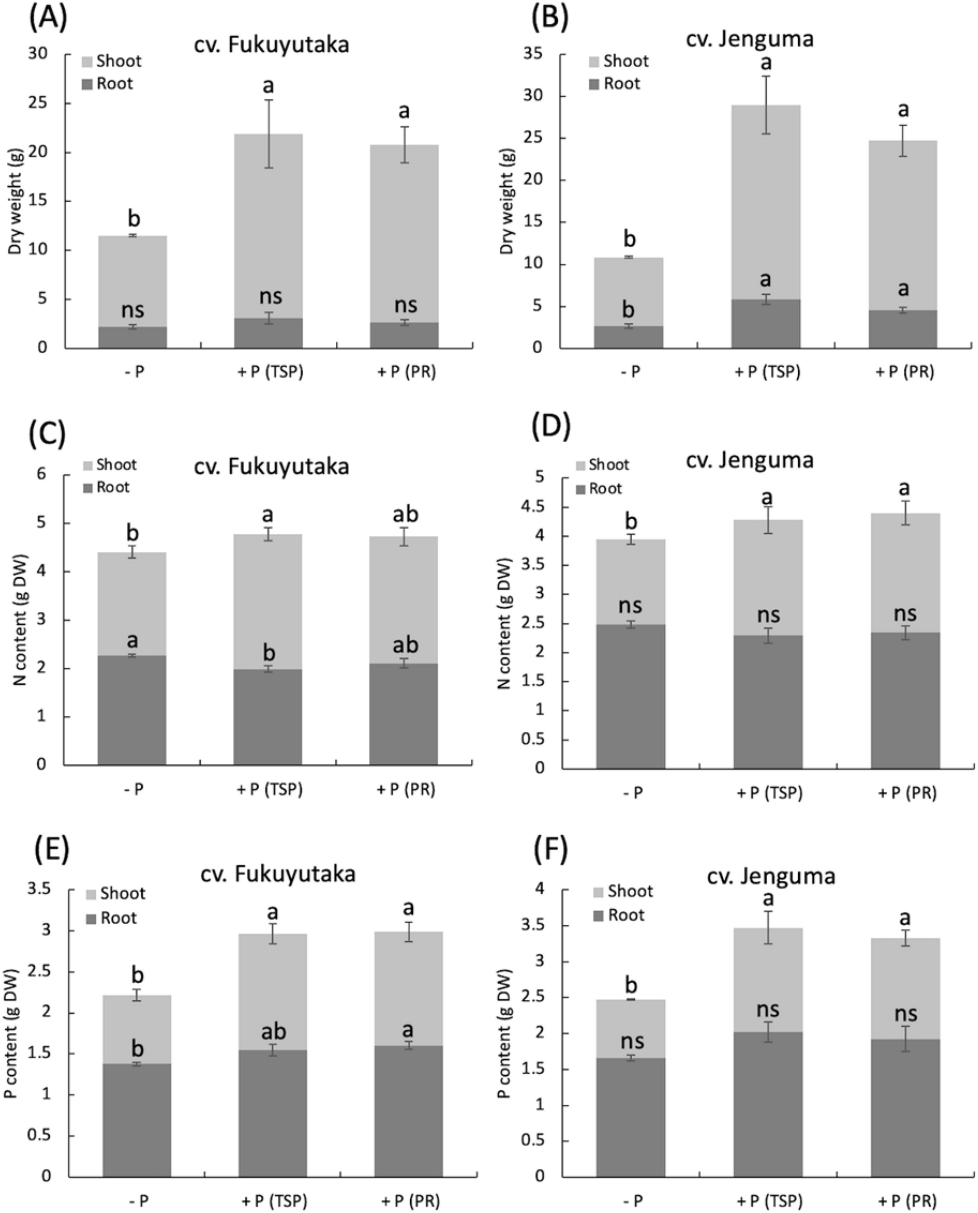
Dry weight, total phosphorus, and nitrogen contents of the shoots and roots of soybean. For each analyzed variable in the shoots and roots, different letters indicate significant mean differences at *p* < 0.05 according to Tukey’s HSD test. **(A)** Effect of P treatments on the dry weight of shoots and roots in the soybean cultivar Fukuyutaka. **(B)** Effect of P treatments on the dry weight of shoots and roots in the soybean cultivar Jeguma. **(C)** Effect of P treatments on the nitrogen content of shoots and roots in the soybean cultivar Fukuyutaka. **(D)** Effect of P treatments on the nitrogen content of shoots and roots in the soybean cultivar Jenguma. **(E)** Effect of P treatments on the phosphorus content of shoots and roots in the soybean cultivar Fukuyutaka. **(F)** Effect of P treatments on the phosphorus content of shoots and roots in the soybean cultivar Jenguma. ns, non-significant difference. P, phosphorus; TSP, triple superphosphate; PR, calcined phosphate rock; DW, dry weight; N, nitrogen; cv., cultivar.

Two-way ANOVA revealed a significant interaction effect between P fertilization and cultivar on dry matter production and N and P contents, so the data are reported separately for each cultivar. Regardless of the P source, P fertilization significantly increased shoot dry weight in both Fukuyutaka and Jenguma, with values at least double those of the control. Both TSP and PR had similar effects on shoot dry weight in both cultivars. The +P(TSP) and +P(PR) treatments significantly increased the root dry weight of Fukuyutaka compared with the -P treatment (**Figures 1A, B**). P fertilization also influenced the nitrogen content of shoots. TSP significantly increased the shoot total N of Fukuyutaka, whereas PR did not. However, both TSP and PR increased the shoot N of Jenguma compared with that of the control. Interestingly, the -P treatment resulted in a significantly greater shoot N content than the TSP treatment, which resulted in the highest shoot N content in Fukuyutaka. P fertilization did not affect the root N content in Jenguma (**Figures 1C, D**). The shoot P content followed a similar trend to that of shoot dry weight, with both TSP and PR significantly increasing shoot P compared with the control in both cultivars. Compared with the control, PR significantly increased the root P content in Fukuyutaka, whereas TSP did not. No significant effect of P fertilization was observed on the root P content in Jenguma (**Figures 1E, F**).

### 3.3 Root nodule number, nitrogen fixation, and nitrogen derived from the atmosphere

**Figure 2:**
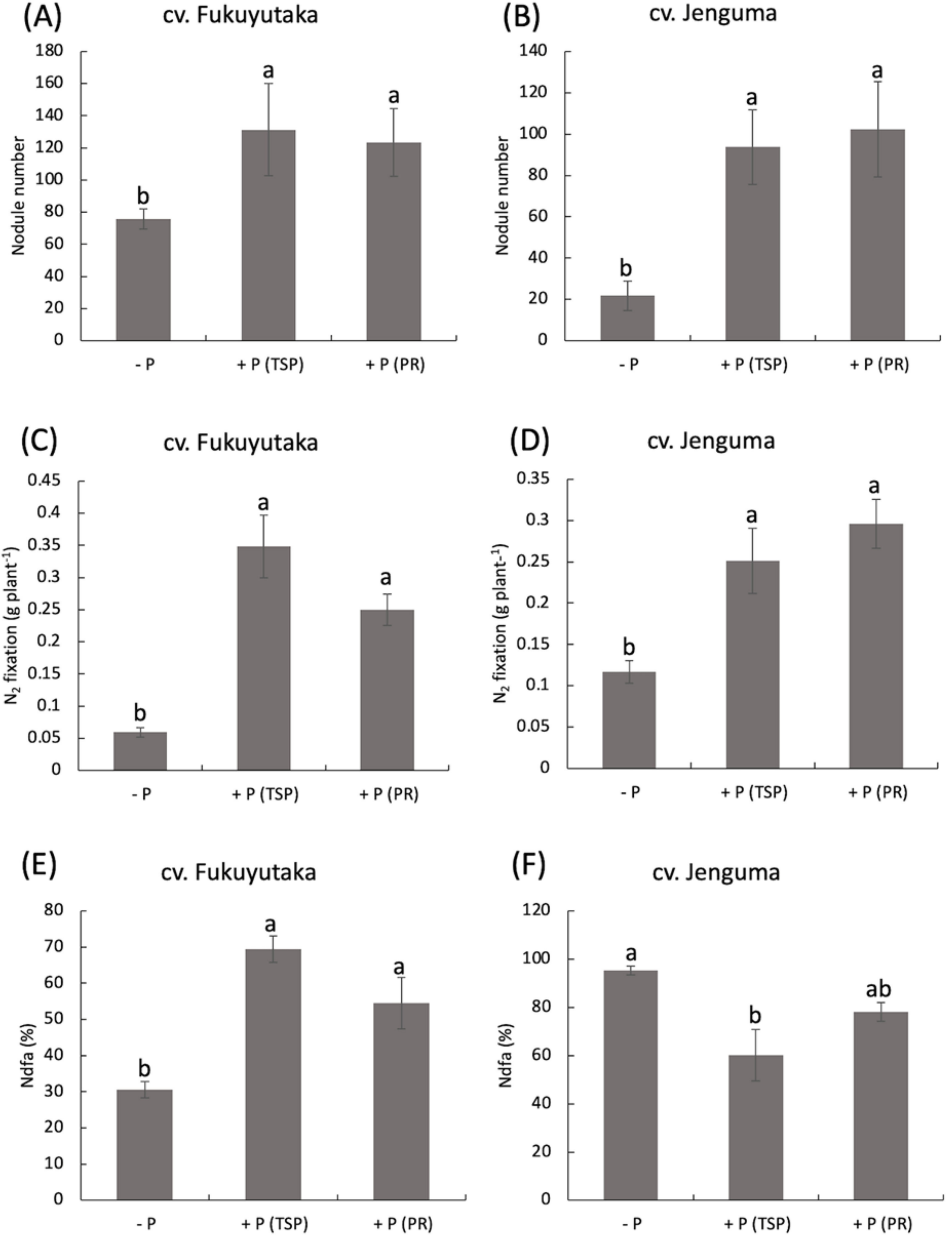
Nodule number, N fixation and Ndfa as affected by P application in soybean. For each analyzed variable, different letters indicate significant mean differences at *p* < 0.05 according to Tukey’s HSD test. **(A)** Effect of P treatments on the nodule number of the soybean cultivar Fukuyutaka. **(B)** Effect of P treatments on the nodule number of the soybean cultivar Jeguma. **(C)** Effect of P treatments on the percentage of nitrogen fixation in the soybean cultivar Fukuyutaka. **(D)** Effect of P treatments on the percentage of nitrogen fixation in the soybean cultivar Jenguma. **(E)** Effect of P treatments on the percentage of nitrogen-derived from atmosphere in the soybean cultivar Fukuyutaka. P, phosphorus; TSP, triple superphosphate; PR, calcined phosphate rock; Ndfa, nitrogen derived from atmosphere; cv., cultivar; N2, atmospheric nitrogen.

P application significantly increased the root nodule number of both cultivars, with a more pronounced effect in Jenguma. In Jenguma, the number of nodules increased fourfold with TSP and fivefold with PR compared with those in the control. The (-P) treatment resulted in only 22 nodules for Jenguma, but TSP and PR application increased this number to 94 and 102 nodules, respectively. In contrast, Fukuyutaka had more nodules (80) than Jenguma (22) under the no-P treatment, with significantly greater nitrogen fixation in both cultivars under the TSP and PR treatments (**Figures 2A, B**). The amount of nitrogen-fixed followed the same trend as the nodule number, with significantly greater values in both cultivars under the TSP and PR treatments (**Figures 2B, C**). For Fukuyutaka, nitrogen fixation increased sevenfold under TSP and fivefold under PR. In Jenguma, the increase was two and a half fold under TSP and threefold under PR (**Figures 2C, D**). The percentage of Ndfa showed a similar trend. In Fukuyutaka, Ndfa increased from 30% to 70% with TSP and 50% with PR. In Jenguma, however, Ndfa decreased to approximately 40% with TSP and to 10% with PR a (**Figures 2E, F**).

### 3.4 Changes in the content of seed protein, lipid, and phosphorus

**Figure 3:**
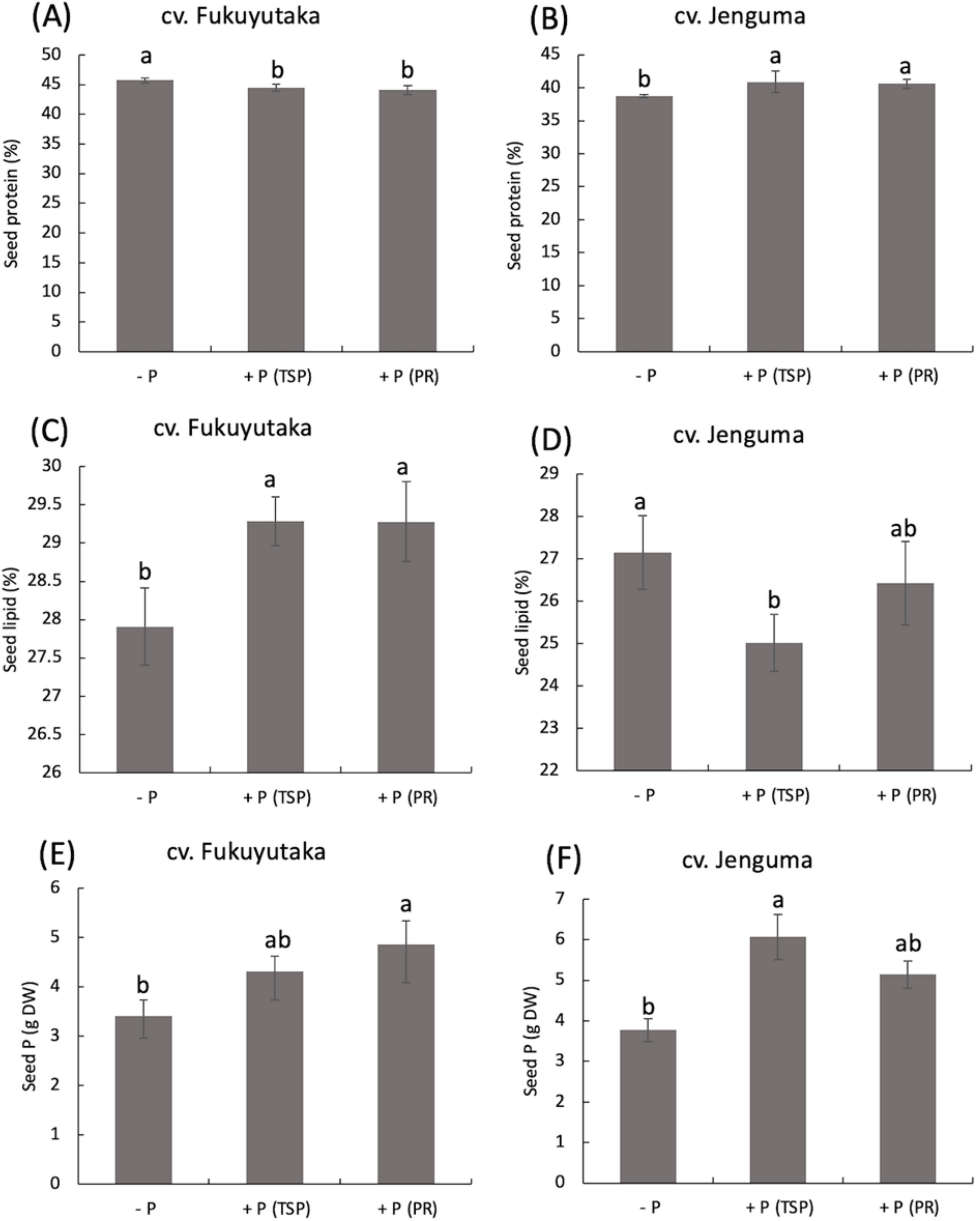
Seed composition of protein, lipid and phosphorus in soybean. For each analyzed variable, different letters indicate significant mean differences at *p* < 0.05 according to Tukey’s HSD test. **(A)** Effect of P treatments on seed protein in the soybean cultivar Fukuyutaka. **(B)** Effect of P treatments on seed protein in the soybean cultivar Jeguma. **(C)** Effect of P treatments on seed lipid in the soybean cultivar Fukuyutaka. **(D)** Effect of P treatments on seed lipid in the soybean cultivar Jenguma. **(E)** Effect of P treatments on seed protein in the soybean cultivar Fukuyutaka. **(F)** Effect of P treatments on seed phosphorus in the soybean cultivar Jenguma. **(F)** Effect of P treatments on the percentage of nitrogen-derived from atmosphere in the soybean cultivar Jenguma. P, phosphorus; TSP, triple superphosphate; PR, calcined phosphate rock; DW, dry weight; cv., cultivar.

P application had contrasting effects on the seed protein content of the two soybean cultivars. In Fukuyutaka, TSP and PR applications significantly decreased the protein percentage compared with that of the -P treatment. In contrast, Jenguma presented a significant increase in the seed protein percentage under both the TSP and PR treatments compared with the -P treatment. Overall, Fukuyutaka contained more protein (33–45%) than Jenguma (38–40%) (**Figures 3A, B**). The lipid percentage of seeds exhibited the opposite trend to that of protein. TSP and PR applications significantly increased the lipid percentage in Fukuyutaka seeds, with both P treatments resulting in a 2% increase. In contrast, TSP application decreased the lipid percentage in Jenguma seeds by 2%, whereas PR maintained the lipid content at a level similar to that of the –P (**Figures 3C, D**). The P contents in the seeds increased under both P applications for both cultivars.

## 4 Discussion

### 4.1 Effects of P fertilization on the growth and yield of soybean

P application, whether in the form of TSP or calcined PR, significantly improved shoot growth, nutrient accumulation, seed yield, and nitrogen, and phosphorus contents across both cultivars, underscoring the crucial role of phosphorus in enhancing both vegetative growth and reproductive success. While both cultivars responded positively to P fertilization, with notable increases in shoot biomass, the effects on root yield and nutrient content varied, highlighting cultivar specific differences in P utilization. These findings align with those of previous studies, such as those of Wang et al. (2009), who reported increased plant dry weight following the overexpression of the APase gene, a mechanism that enhances the ability of plants to acquire or remobilize inorganic P from organic sources. These findings indicate that improving P availability could have similar beneficial effects in our study. However, the differential response in terms of root yield highlights the need for further investigation into cultivar specific P utilization mechanisms. Under P deficient conditions, P application improved soybean shoot growth (He et al., 2019), which, in turn, increased the plant area, increasing light interception for photosynthesis. The increase in shoot dry weight resulting from P fertilization in both cultivars likely contributed to the observed higher seed yield, demonstrating how P availability enhances both vegetative growth and reproductive success (Xu et al., 2024; Kakiuchi, 2018). The similar positive effects of both applied P sources indicate that calcined PR is an effective amendment for enhancing soybean growth and seed yield. In this study, soybean cultivars from Japanese (Asian) and African origins were used, and the positive response observed in both suggests that PR can effectively increase soybean yields across different origins. Previous studies have also demonstrated that the direct application of non-calcined raw phosphate rock can replace chemical phosphorus fertilizers, such as TSP, to improve crop yields, including those of lowland rice (Nakamura et al., 2013; 2016). Additionally, low grade raw phosphate rock has been found to increase cowpea, groundnut, and wheat yields, particularly when combined with plant growth-promoting rhizobacteria (Saleem et al., 2013; Iseki et al., 2024). The present study provides evidence that calcined PR is an additional source of P that can improve the yield of leguminous crops, such as soybeans.

In addition to its positive influence on soybean aboveground biomass, P fertilization led to significant increases in both the N and P contents in the shoots of Fukuyutaka and in the P content in Jenguma, again highlighting cultivar specific responses to fertilization. To produce high quality seeds, soybean plants require substantial amounts of N and P. As leguminous crops, most of the soybean N is obtained via N_2_ fixation in root nodules, whereas P is sourced primarily from the soil (Faozi et al., 2019). Nodule formation and nitrogen fixation are energy intensive processes, and P application plays a crucial role in providing the energy needed for these processes while also increasing the uptake of both N and P. Additionally, P and N are essential for photosynthesis and overall plant development. Bindraban et al. (2020) noted that low P concentrations reduce the maximum photosynthetic rate and leaf N concentration. Photosynthesis increases as the leaf P concentration increases, leading to maximum biomass and grain yield when the soybean leaf P concentration is between 0.2% and 0.3% by weight (Singh et al., 2018). Based on these findings, we suggest that P fertilization primarily improves shoot growth, likely through increased nutrient uptake. The increase in shoot growth due to P fertilization may suggest that P availability is critical in improving soybean yield, especially in soils with limited phosphorus. The lack of significant differences in root dry weight across treatments in both cultivars may indicate that extensive root growth is not required under favorable soil conditions. The impact of P on root growth may not only be limited to biomass but also influence crucial processes such as nitrogen fixation. While P fertilization primarily enhanced shoot growth, its effects on root development were also significant, particularly in terms of supporting symbiotic nitrogen fixation. This aligns with previous research indicating that adequate P is critical for energy intensive processes such as nodule formation and nitrogen fixation (Somado et al., 2006).

### 4.2 Symbiotic nitrogen fixation improved with increasing root nodule number

P fertilization, through both TSP and PR, stimulated root nodule formation and nitrogen fixation, demonstrating that phosphorus availability is vital for energy-intensive processes such as nodule development, which supports improved N_2_ fixation in soybeans. Similar findings were reported by Somado et al. (2006), where PR and TSP applications equally increased the biomass and %Ndfa in the legume *Crotalaria micans*, further highlighting the importance of P amendments to support nitrogen fixation. The required P can be supplied by PR instead of the expensive chemical fertilizer TSP. This positive effect of the PR may stem from its enhanced solubility following calcination (Nakamura et al., 2015). As Taliman et al. (2019) reported, appropriate P fertilization can regulate the growth and development of soybean nodules, thereby increasing N_2_ fixation and improving plant growth and yield. Although the specific mechanisms through which P affects nodule nitrogen fixation in soybeans remain unclear (Li et al., 2022b), previous studies have shown that low P concentrations in soil reduce the energy costs for nodule formation, function, and nitrogen fixation in legumes (Vardien et al., 2016). The importance of phosphorus (P) for nodulation and nitrogen fixation has been highlighted in previous studies, which have shown that functional nodules typically contain two to three times more P than other plant organs (Sa and Israel, 1991; Qin et al., 2012; Vauclare et al., 2013). Furthermore, Zhong et al. (2023) reported that leguminous plants grown under high P conditions produced significantly more nodules than those grown under low-P conditions, suggesting that increased P availability promotes nodule formation and, in turn, enhances nitrogen fixation capacity. Therefore, the observed improvement in nitrogen fixation in this study may be attributed to the increase in root nodule number, which was stimulated by the application of both P fertilizers. Although the nodule mass was not recorded in this study, the parallel increase in nodule number and N_2_ fixation suggested that the formed nodules were mature and large enough to initiate nitrogenase activity. The overall positive effect of P application on nitrogen fixation and growth improvements set the stage for changes at the seed level, as seen in the alterations in seed composition.

### 4.3 Variations in seed composition by component, P source, and cultivar

P fertilization influenced seed composition, resulting in small but significant changes, such as increased protein content in Jenguma and increased lipid content in Fukuyutaka, due to N and P increments in shoot, indicating that genotype-specific responses may affect both agricultural productivity and food quality. This inverse relationship between protein and lipid concentrations is consistent with findings from previous research (Allen and Young, 2013; Kambhampati et al., 2020). P fertilization was found to impact seed composition, although the changes are often moderate and inconsistent (Yin et al., 2016). The quality of soybean seeds is influenced by genetic and environmental factors, such as genotype, cultivar maturity, temperature, drought, and soil nutrients (Bellaloui et al., 2011). Bellaloui et al. (2015) reported that later planting during the season results in higher protein but lower oil concentrations. Environmental factors, such as soil moisture and temperature, in addition to differences in nutrient accumulation in seeds and leaves, could help explain these variations in protein and oil concentrations. Given that this study was conducted in a greenhouse with controlled growth conditions, it is possible that the two soybean varieties responded differently to the set temperature. This could have influenced the soil moisture content in distinct ways due to variations in growth patterns, potentially accounting for differences observed in the seed protein and lipid contents following P fertilization. On the other hand, the increase in the seed P content of both Fukuyutaka and Jenguma due to P fertilization may improve soybean seed quality nutritionally, as phytate, the primary form of P in seeds, offers health benefits to humans, including anticarcinogenic properties, potent antioxidant activity, and inhibition of kidney stone formation (Gemede, 2014; Bindraban et al., 2020).

## 5 Conclusion

This study highlights the crucial role of phosphorus fertilization in enhancing soybean growth, yield, and seed quality in phosphorus-deficient soils, such as those common in sub-Saharan Africa. Both triple superphosphate (TSP) and calcined phosphate rock (PR) significantly improved these parameters, with calcined PR offering a cost-effective alternative to TSP. The positive effects of phosphorus on nitrogen fixation further support its role in sustainable soybean cultivation. While this study focused on two cultivars, future research should include a wider range of soybean genotypes to better understand the interactions between genotype and phosphorus fertilizer source. Overall, calcined PR represents a promising solution to improve soil fertility and crop yields for smallholder farmers in sub-Saharan Africa.

## Data Availability

The original contributions presented in the study are included in the article/**Supplementary Material**. Further inquiries can be directed to the corresponding author.
