# Supplementary material for: Calcined low-grade phosphate rock fertilization enhances nitrogen fixation, yield, and grain quality in soybeans
Source: Front Plant Sci. 2025 Jun 19;16:1581961. doi: 10.3389/fpls.2025.1581961 (PMC12222267; doi:10.3389/fpls.2025.1581961)
Supplement: Supplementary file 1 [file Table1.docx]

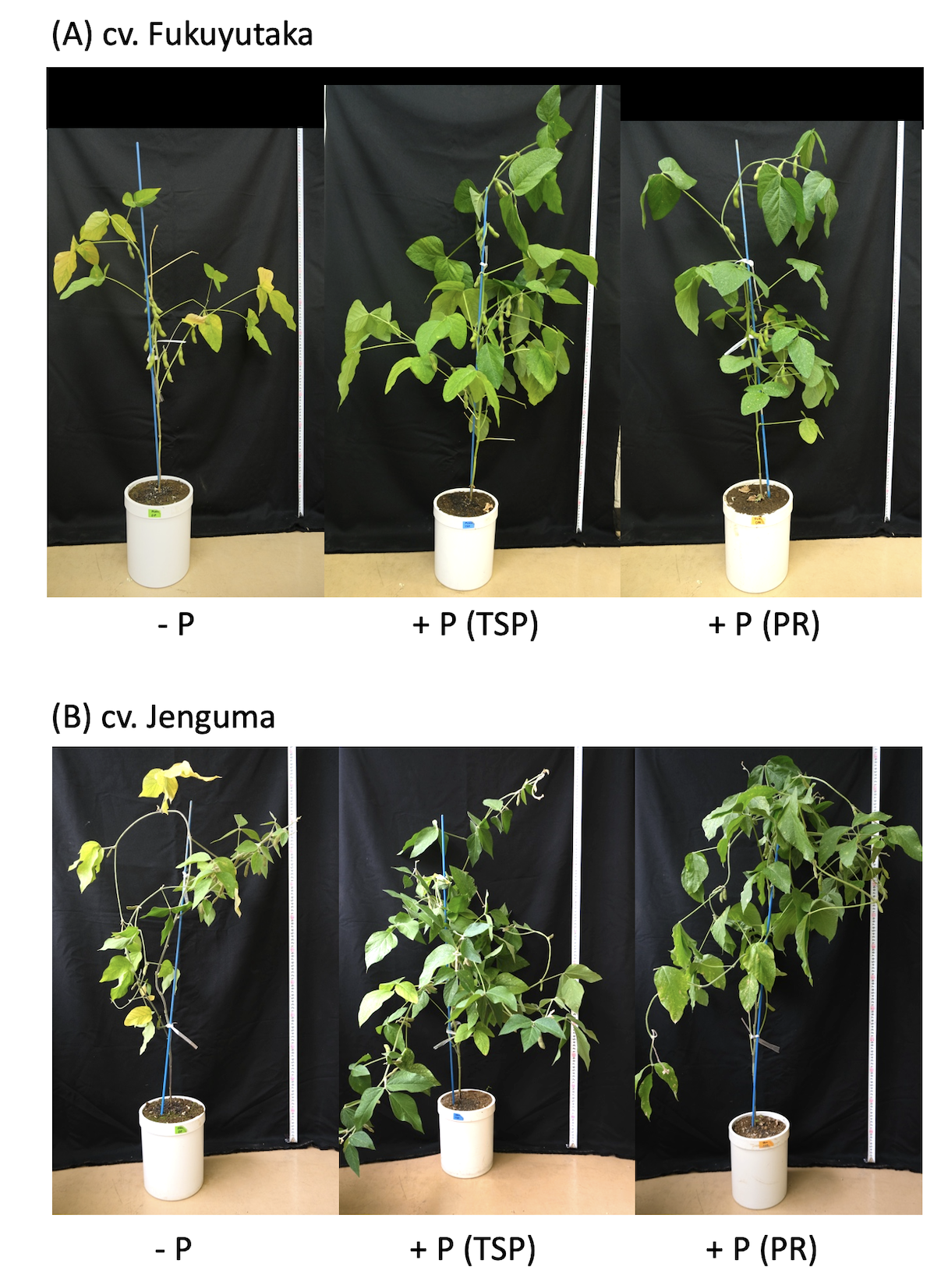


Supplementary Fig. S1. Growth phenotype of the cultivars Fukuyutaka (A) and Jenguma (B) following P application

P: phosphorus, TSP: triple superphosphate, PR: calcined phosphate rock
